# The Metabolic Basis of Immune Dysfunction Following Sepsis and Trauma

**DOI:** 10.3389/fimmu.2020.01043

**Published:** 2020-05-29

**Authors:** Margaret A. McBride, Allison M. Owen, Cody L. Stothers, Antonio Hernandez, Liming Luan, Katherine R. Burelbach, Tazeen K. Patil, Julia K. Bohannon, Edward R. Sherwood, Naeem K. Patil

**Affiliations:** ^1^Department of Pathology, Microbiology and Immunology, Vanderbilt University Medical Center, Nashville, TN, United States; ^2^Department of Anesthesiology, Vanderbilt University Medical Center, Nashville, TN, United States

**Keywords:** sepsis, infection, trauma, trained immunity, mitochondria, metabolic reprogramming

## Abstract

Critically ill, severely injured and high-risk surgical patients are vulnerable to secondary infections during hospitalization and after hospital discharge. Studies show that the mitochondrial function and oxidative metabolism of monocytes and macrophages are impaired during sepsis. Alternatively, treatment with microbe-derived ligands, such as monophosphoryl lipid A (MPLA), peptidoglycan, or β-glucan, that interact with toll-like receptors and other pattern recognition receptors on leukocytes induces a state of innate immune memory that confers broad-spectrum resistance to infection with common hospital-acquired pathogens. Priming of macrophages with MPLA, CPG oligodeoxynucleotides (CpG ODN), or β-glucan induces a macrophage metabolic phenotype characterized by mitochondrial biogenesis and increased oxidative metabolism in parallel with increased glycolysis, cell size and granularity, augmented phagocytosis, heightened respiratory burst functions, and more effective killing of microbes. The mitochondrion is a bioenergetic organelle that not only contributes to energy supply, biosynthesis, and cellular redox functions but serves as a platform for regulating innate immunological functions such as production of reactive oxygen species (ROS) and regulatory intermediates. This review will define current knowledge of leukocyte metabolic dysfunction during and after sepsis and trauma. We will further discuss therapeutic strategies that target leukocyte mitochondrial function and might have value in preventing or reversing sepsis- and trauma-induced immune dysfunction.

## Introduction

Serious infection is a major threat to critically ill patients and frequently precipitates sepsis, a complex disease spectrum that includes systemic inflammation and organ dysfunction. As such, sepsis is the leading cause of death in non-cardiac intensive care units (ICU) and accounts for 40% of ICU expenditures (1). Early investigators postulated that systemic inflammation was the underlying factor driving the pathogenesis of sepsis and septic shock (2–4). High concentrations of pro-inflammatory mediators such as tumor necrosis factor, IL-1, and platelet activating factor were present in plasma and fluids of septic animals and humans (3, 5). Blockade of pro-inflammatory mediators in experimental animals attenuated or prevented the development of septic shock (6, 7). Those observations prompted clinical trials aimed at blocking cytokine and non-cytokine mediators of inflammation, which were not successful at improving survival in patients with severe sepsis or septic shock (8). Specifically, a trial of anakinra, a recombinant IL-1 receptor antagonist, was not found to be effective in improving mortality in sepsis (9). However, a subgroup analysis found that the use of anakinra improved survival in patients with concurrent hepatobiliary dysfunction and disseminated intravascular coagulation, which are specific features of macrophage activation syndrome (10). Therefore, subgroup analysis of diverse sepsis patients for underlying conditions needs to be considered in studies evaluating different sepsis treatments to better understand the therapeutic benefit in different sub-populations of sepsis patients. Later investigations showed that septic patients had impaired innate and adaptive antimicrobial immunity, which resulted in their inability to control primary and secondary infections. Likewise, patients that survive sepsis and severe trauma have long-term physical and cognitive disabilities and frequently require readmission to the hospital due to recurrent infections (11). Research indicates that the septic or severely injured host responds to severe inflammation by activating anti-inflammatory pathways to mitigate further inflammatory injury. Among those pathways are increased production of anti-inflammatory cytokines such as IL-10 and transforming growth factor-β (TGFβ) and upregulation of checkpoint inhibitors such as PD-1, CTLA-4, BTLA, and PDL1 by leukocytes (12, 13). Other investigators have shown large-scale apoptosis and dysfunction of lymphocytes and the proliferation of myeloid-derived suppressor cells, which act to suppress innate and adaptive antimicrobial responses (14, 15). Most recently, the concept of metabolic dysfunction has emerged as a factor underlying impaired function of the innate and adaptive immune systems of septic and severely injured patients. This paper will review current knowledge of leukocyte metabolic dysfunction in the setting of sepsis and severe injury and discuss interventions to improve leukocyte metabolism and function.

## Overview of Sepsis-Induced Mitochondrial Dysfunction

Glycolysis and mitochondrial oxidative phosphorylation form the backbone of cellular metabolism. Glucose is primarily metabolized to pyruvate through glycolysis, along with a net generation of two ATP molecules. Cells transport pyruvate into mitochondria where it is metabolized to acetyl-CoA via the enzymatic action of the pyruvate dehydrogenase complex (PDH). Acetyl- CoA is metabolized through a series of enzymatic reactions in the mitochondrial tricarboxylic acid (TCA) cycle to produce reducing intermediates including NADH and FADH2, which feed electrons into the TCA cycle-linked electron transport chain (ETC). Optimal flow of electrons through ETC complexes (I-IV) is required for maintenance of mitochondrial membrane potential and proton gradient, which ultimately facilitate ATP generation (16). Recent studies show that mitochondria not only generate adenosine triphosphate (ATP), but also are intricately involved in cellular signaling pathways that regulate calcium homeostasis, reactive oxygen species (ROS) generation, redox signaling, and maintenance of immune cell competence, all of which are critical for our survival (17–19).

The 3rd International Consensus Conference defined sepsis as organ dysfunction caused by a dysregulated host response to infection (20). Evidence indicates that mitochondrial dysfunction is a key player in induction and propagation of sepsis-induced organ injury, which is demonstrated in both animal and human studies (21, 22). Brealey et al., were among the first to demonstrate that sepsis leads to significant impairment of skeletal muscle mitochondrial ETC activity (specifically complex I), which correlates with the severity of septic shock in humans (23). Furthermore, decreased skeletal muscle ATP concentrations were predictive of increased mortality among sepsis patients. A clinical study by Matkovich et al., showed a striking 43% decline in levels of mRNA that encode proteins involved in mitochondrial TCA cycle and ETC complexes in the hearts of septic patients (24). Numerous animal studies also demonstrate a role for mitochondrial dysfunction in sepsis pathology. Using animal models, sepsis has been shown to cause a significant impairment of mitochondrial function in multiple organs including heart, kidney, liver, and skeletal muscle (25–28). Although these studies demonstrate a role for mitochondrial dysfunction in sepsis pathology, discrepancies in various studies also show a highly variable mitochondrial function in multiple organs depending on the sepsis model used, severity of sepsis induced, time course studied, and methodology used for measurement of mitochondrial function (29). Therefore, there remains some controversy in the field as to whether mitochondria are the actual initiators or concurrent amplifiers of organ dysfunction during sepsis (29).

## Sepsis-Induced Mitochondrial Dysfunction in Leukocytes

Recent studies demonstrate that sepsis-induced impairment of leukocyte mitochondrial function contributes to impaired antimicrobial immune responses and increased susceptibility to secondary infections (30, 31). The majority of the studies implicating a role for sepsis-induced leukocyte mitochondrial dysfunction used Peripheral Blood Mononuclear Cells (PBMCs) isolated from septic patients (summarized in Table 1). Adrie et al., demonstrated significant sepsis-induced depolarization of mitochondrial membrane potential and increased expression of cell death markers in peripheral blood monocytes. Eventual non-survivors demonstrated higher depolarization of the mitochondrial membrane as compared to survivors (32). Other studies showed a reduction in mitochondrial respiration in the presence of high ADP and P_i_ (also known as state 3 respiration), ATP synthase complex activity and mitochondrial spare respiratory capacity in PBMCs from sepsis patients (33, 34, 39). Reduced mitochondrial respiration in leukocytes was associated with increased incidence of organ failure (34). Garrabou et al., demonstrated a significant impairment of mitochondrial ETC complexes I, III, and IV in PBMCs of patients with confirmed systemic infection but without septic shock (35).

**Table 1:** Summary of clinical studies showing sepsis-induced alterations in leukocyte mitochondrial function.

**References**	**Sepsis definition and patient age**	**Sample analyzed**	**Time of sample collection after sepsis diagnosis**	**Major alterations in mitochondrial function (as compared to controls)**
Adrie et al. (32)	Severe sepsis and septic shock (>18 years)	PBMC	- Within 72 h - Between 7th and 10th day	- Increased membrane depolarization - Increased cell death markers
Belikova et al. (33)	Severe sepsis and septic shock (>18 years)	PBMC	- Within 48 h of ICU admission	- Reduced ADP-stimulated state 3 respiration and increased basal oxygen consumption
Japiassu et al. (34)	Septic shock (>18 years)	PBMC	- Within 48 h	- Reduced ADP-stimulated state 3 respiration and ATP synthase activity
Garrabou et al. (35)	SIRS with infection (no septic shock)	PBMC	- Exact time point not mentioned	- Decreased activities of ETC complexes I, III, and IV - Unaltered mitochondrial mass
Sjovall et al. (36)	Severe sepsis and septic shock (>18 years)	PBMC	- Within 48 h - Days 3–4 - Days 6–7	- Basal respiration and ETC complex I, II, and IV activities increased over time up to day 7
Weiss et al. (37) **(pediatric study)**	Septic shock with organ failure (<18 years)	PBMC	- Within 48 h - Days 5–7	- Unaltered basal and ATP linked respiration on days 1–2 - Spare respiratory capacity (SRC) decreased on days 1–2 - SRC recovered over days 5–7
Cheng et al. (31)	LPS infusion in healthy volunteers Bacterial and fungal sepsis patients (>18 years)	PBMC and monocytes	- LPS infusion for 4 h - Within 24 h for septic patients	- Decreased oxygen consumption in all models - Both glycolytic capacity and mitochondrial function impaired in septic PBMCs - Impaired ability to respond to a second stimulus
Merz et al. (38)	Septic shock (> 18 years)	Monocytes	−24 and 48 h - At shock resolution	- ETC complex I, IV, and ATP synthase activities elevated - No difference in ATP content
Jang et al. (39)	Sepsis and septic shock (>18 years)	PBMC	- Within 24 h	- Decreased ATP-linked respiration and reduced uncoupled complex I activity, and no differences in ETC complex II and IV activities. - Decreased spare respiratory capacity
Kraft et al. (40)	Sepsis with evidence of organ injury (>18 years)	PBMC	- Days 1, 3, and 5	- Reduced mitochondrial DNA and mitochondrial biogenesis - Increased plasma D-loop indicating mitochondrial damage - Alterations normalized over a week with patients' recovery
Weiss et al. (41) **(pediatric study)**	Sepsis and septic shock (<18 years)	PBMC	- Days 1–2, 3–5 and 8–14	- Decreased spare respiratory capacity (SRC) and increased mitochondrial content on days 1–2 - SRC recovered over time as patients improved over 14 days. - Low SRC associated with residual organ injury at day 14.
Weiss et al. (42) **(pediatric study)**	Severe sepsis and septic shock (<18 years)	PBMC	- Within hours - Days 3–5 and 8–14	- Decreased mitochondrial respiration observed in those septic PBMCs which showed reduced LPS-induced TNF-α and HLA-DR expression.
Clere-Jehl et al. (43)	Septic shock (<18 years)	PBMC	- Within 12 hours of noradrenaline start	- Increased basal and maximal respiratory capacity - Lower ATP synthase activity

In a major study, Cheng et al., showed that both bacterial and fungal sepsis leads to a shift in cellular metabolism toward glycolysis (Warburg effect), and leukocytes isolated from septic patients, as well as those treated with lipopolysaccharide (LPS), demonstrated a reduced oxygen consumption capacity signifying mitochondrial defects (31, 44). Furthermore, these metabolic defects were associated with impaired ability of leukocytes to produce pro-inflammatory cytokines in response to a secondary stimulus, which the authors refer to as a state of immunoparalysis (31). A study by Kraft et al., brings to light an important observation that effective reversal of the initial sepsis-induced leukocyte mitochondrial damage via early activation of mitochondrial biogenesis improved clinical outcomes among septic patients (40). They showed that mRNA levels of genes related to mitochondrial biogenesis, including PGC-1α, NRF1, and TFAM, were significantly reduced 1 day after the initiation of sepsis along with a decrease in mitochondrial DNA copy number. Recovery of these parameters was paralleled by improved clinical outcome and discharge from the ICU over a 1 week period (40). In multiple pediatric studies using PBMCs, Weiss et al., demonstrated that sepsis leads to a significant decrease in mitochondrial respiration and spare respiratory capacity implying a decreased bioenergetic reserve and mitochondrial dysfunction (37, 41, 42).

In contrast to these studies demonstrating sepsis-induced impairment of mitochondrial respiration, some studies show unaffected or increased mitochondrial respiration. Using PBMCs and monocytes from patients with severe sepsis and septic shock, Sjovall et al., and Merz et al., showed a significant increase in activities of mitochondrial ETC complexes I, II, and IV and did not observe a difference in these parameters among survivors vs. non-survivors (36, 38). In line with these studies, Clere-Jehl et al., showed that sepsis leads to a significant increase in mitochondrial respiratory capacity of PBMCs (43). However, mitochondrial respiration was impaired upon suspending the PBMCs in septic plasma, implying a role for a soluble plasma factor, which the authors attributed to a high level of HMGB1 (43). The contrasting findings might be attributed to the vast heterogeneity in sepsis patient populations, differing time points selected for measurements and underlying co-morbidities. Leukocyte-specific mitochondrial function in freshly isolated systemic immune cells has not been assessed in animal models.

In summary, the majority of studies implicate mitochondrial dysfunction as an important contributor toward sepsis-induced leukocyte and organ dysfunction. Importantly, early recovery of mitochondrial function correlates positively with improved clinical outcomes in septic patients (40, 45). Therefore, therapies targeting recovery of mitochondrial function hold potential for reversing leukocyte dysfunction during sepsis. Agents that target the AMP kinase pathway, such as AICAR (5-aminoimidazole-4-carboxamide ribonucleotide), or the mTOR signaling pathway, such as metformin, could provide benefit. Recent studies demonstrate that activation of pattern recognition receptors of innate leukocytes, especially monocytes and macrophages, augments mitochondrial function and rewires mitochondrial metabolism leading to accumulation of specific TCA cycle intermediates such as citrate, itaconate, succinate, fumarate, and others. Prophylactic treatment with TLR4 agonists can protect against severe infections for up to 14 days (46–48). That benefit is due, in part, to heightened mitochondrial and antimicrobial functions in macrophages Therefore, TLR agonist-induced mitochondrial metabolic reprogramming in innate leukocytes is associated with the generation of distinct innate immune memory. Mitochondrial reprogramming and innate immune memory are now being widely investigated as novel strategies for developing mitochondria-targeted therapies for protection against infections and sepsis in critically ill patients.

## The Impact of Trauma on Leukocyte Metabolism

Although similar to sepsis, trauma provides a different set of signals to the immune system. While infection and sepsis can be a complication of trauma, the direct impact of trauma on immune system function is generated through tissue injury, inflammation, and tissue ischemia and reperfusion (49, 50). The effect of trauma on immune function is variable and largely dependent on the severity of injury (51, 52). The release of endogenous cell products, such as mitochondrial DNA, oxidized phospholipids, and ATP can activate toll-like receptors and inflammasomes to precipitate immune system activation (53, 54). Excessive or inappropriate immune system activation following major trauma could lead to immune dysfunction. Impairment of neutrophil and monocyte chemotaxis and antimicrobial functions have been described (55–57) as have alterations in lymphocyte function (58). However, little is known about the impact of major trauma on the metabolic state of leukocytes, which raises an area for research.

## Potential Therapeutic Strategies Targeting Leukocyte Mitochondrial Function During Sepsis and Trauma

Effective mitochondrial biogenesis requires a coordinated action of complex intracellular pathways including both nuclear and mitochondrial genome encoded proteins (59, 60). PGC-1α is recognized as one of the most important and inducible transcription factor that drives mitochondrial biogenesis in response to external stimuli for maintaining mitochondrial homeostasis (61). The activity of PGC-1α is regulated by post-translational modifications. Sirtuin 1 (SIRT1)-induced deacetylation and adenosine monophosphate-activated protein kinase (AMPK)-induced phosphorylation are known to activate PGC-1α (62). Along with PGC-1α, other cellular transcription factors and mediators, including NRF1 and NRF2, PGC-1β, TFAM, ERRα, CREB, also play an important role in regulating mitochondrial biogenesis (63). The following section will discuss some of the promising therapeutic strategies targeting augmentation of mitochondrial biogenesis, which could be applicable for protecting or restoring leukocyte mitochondrial function during sepsis and trauma.

### Pharmacological Agents Targeting Mitochondrial Biogenesis and Function

Studies included in this section are summarized in Table 2.

**Table 2:** Pharmacologic agents targeting mitochondrial biogenesis and function.

**Agent class**	**Specific agent**	**References**	**Model**	**Effect**
AMPK activity enhancer	AICAR	Canto et al. (64)	Mouse	- Reduced acetylation of PGC1α - Induced expression of PGC1α-target genes in skeletal muscle
		Inata et al. (65)	Mouse CLP	- Protected against cardiac architecture derangement and dysfunction
		Hall et al. (66)	Mouse endotoxemia	- Protected against loss in muscle mass
		Escobar et al. (67)	Mouse CLP	- Reduced pro-inflammatory cytokines - Reduced kidney and liver injury markers
	Metformin	Wang et al. (68)	Mice fed high fat diet	- Improved hepatic mitochondrial complex activity and mitochondrial density in AMPK-dependent manner
		Detaille et al. (69)	HMEC-1 (human immortalized endothelial cell line)	- Inhibited of mitochondrial complex I leading to modulation of the cellular AMP/ATP ratio to activate AMPK
		Meng et al. (70)	Hepa1–6 (mouse hepatoma cell line)	- Activated AMPK via increased phosphorylation of AMPKα at Thr-172
		Suwa et al. (71)	Rats	- Increased PGC-1α expression and mitochondrial biogenesis in skeletal muscle
		Tzanavari et al. (72)	Mouse endotoxemia	- Rescued cardiac dysfunction - Increased ATP synthesis - Reduced inflammatory markers
		Vaez et al. (73)	Isolated rat hearts exposed to LPS	- Activated AMPK - Decreased TLR4 activity - Improved cardiac function
		Vaez et al. (74)	Rat endotoxemia	- Activated AMPK in lung tissue - Reduced inflammatory cell infiltrate in alveolar walls
		Vaez et al. (75)	Rat endotoxemia	- Activated AMPK in cardiac tissue - Decreased myocardial TLR4 - Improved cardiac function
		Tang et al. (76)	Mouse CLP	- Decreased brain edema, preserved BBB, improved cognitive function, improved survival
		Liang et al. (77)	Metanalysis of cohort studies	- Preadmission metformin use was associated with decrease mortality in patients with sepsis and DM
	5HT	Freire-Garabal et al. (78)	Isolated mouse peritoneal macrophages	- Augmented phagocytic capacity of peritoneal macrophages
		Mikulski et al. (79)	Isolated mouse alveolar macrophages	- Increased expression of MCP-1(CCL2)
PPAR activators	Rosiglitazone	Drosatos et al. (80)	Mouse endotoxemia	- Protected mitochondria, reduced cardiac dysfunction, and improved survival
	Pioglitazone	Tsujimura et al. (81)	Mouse CLP	- Reduced inflammation and improved survival
		Majer et al. (82)	Mouse *Candida albicans* sepsis	- Reduced renal pathology and improved survival
	15d-PGJ(2) and	Zingarelli et al. (83)	Rat CLP	- Reduced inflammation, neutrophil infiltration in lung, colon, and liver, hypotension, and improved survival
	Ciglitazone 15d-PGJ(2) and Troglitazone	Maggi et al. (84)	RAW 264.7 cells and CD-1 mouse peritoneal macrophages	- Reduced iNOS, COX-2, IL-1 in cells treated with LPS and IFNγ
	15-PGJ(2)	Guyton et al. (85)	Isolated rat peritoneal macrophages	- Inhibited LPS-induced peritoneal macrophage inflammatory mediators
	15-PGJ(2) Troglitazone	Guyton et al. (86)	Isolated rat peritoneal macrophages	−15-PGJ(2) inhibited LPS, E. coli, and S. aureus-induced NO and TXA - Troglitazone inhibited TXA synthesis in each condition
	Fenofibrate	Tancevski et al. (87)	Murine *Salmonella typhimurium* sepsis	- Reduced pro-inflammatory cytokines, increased neutrophil recruitment, augmented bacterial clearance, improved survival - These effects were independent of PPARα
		Cree et al. (88)	Clinical trial of pediatric burn patients	- Increased hepatic mitochondrial ATP, maintenance of cytochrome C oxidase and citrate synthase activity - Improved insulin sensitivity
	Clofribrate	Crisafulli and Cuzzocrea (89)	Isolated mouse peritoneal macrophages	- Reduced LPS/IFN-γ induced pro-inflammatory cytokine production
PDE inhibitors	Milrinone	Barton et al. (90)	Pediatric sepsis clinical trial	- Increased cardiac index, stroke volume index, and oxygen delivery - Decreased systemic vascular resistance
	Ro 20-1724	Carcillo et al. (91)	Rat endotoxemia	- Improved renal function and survival
		Thomas et al. (92)	Rat endotoxemia	- Protected cardiac contractility and function
	Rolipram	Holthoff et al. (93)	Mouse CLP	- Improved renal blood flow, protected renal microcirculation, improved GFR and renal function
		Sims et al. (94)	Rat pup CLP	- Improved renal, cardiac function, and survival
		Sanz et al. (95)	Rat endotoxemia	- Reduced leukocyte-endothelial interactions
	Rolipram and Roflumilast	Schick et al. (96)	Rat endotoxemia	- Reduced capillary leakage - Stabilized endothelial barrier
	Rolipram	Wollborn et al. (97)	Rat endotoxemia	- Improved hepatic microcirculation and protects liver architecture
	Cilostazol	Zuo et al. (98)	HUVEC	- Induced mitochondrial biogenesis (increased ATP mitochondrial DNA, cytochrome B, and mitochondrial mass) through PGC1α
	Rolipram	Ding et al. (99)	Mouse renal fibrosis by unilateral ureteral obstruction	- Increased mitochondrial biogenesis and PGC1α expression
Natural products	Resveratrol	Biala et al. (100)	Transgenic rat model of heart failure	- Increased PGC-1α, NRF1, NRF2 and Tfam, and mitochondrial biogenesis
		Wang et al. (101)	Rat CLP	- Inhibited of NFκB - Decreased kidney injury - Increased survival
		Luo et al. (102)	Rat CLP	- Decreased renal tubular pathology and proinflammatory cytokines
		Wang et al. (103)	Young rat CLP	- Activated NRF2 - Protects from kidney injury
		Shang et al. (104)	Rat LPS peritonitis	- Protected myocardium and decreased inflammatory markers
		Martin et al. (105)	*Ex-vivo* equine leukocytes	- Did not increase antimicrobial functions - Did not alter cytokine profiles
	ECGC	Valenti et al. (106)	Human Lymphoblasts and fibroblasts	- Increased SIRT1 and PGC1α - Increased mitochondrial complex activities and oxidative phosphorylation efficiently
		Chiou et al. (107)	Mouse endotoxemia	- Activated NRF2 via direct interaction with KEAP1 - Reduced LPS-induced TLR4 activation
		Wang et al. (108)	Mouse endotoxemia	- Protected against acute lung injury - Decreased proinflammatory cytokines
		Wheeler et al. (109)	Mouse and rat CLP	- Decreased hypotension - Improved survival
	Daidzein and Genistein (Phytoestrogens)	Cederroth et al. (110)	Mouse	- Diet containing both compounds increased PGC-1α expression
	Daidzein	Parida et al. (111)	Mouse CLP	- Suppressed lung injury, decreased bacterial load
	Genistein	Yi et al. (112)	Mouse endotoxemia	- Suppressed proinflammatory cytokines from endothelial cells

#### Modulators of AMPK Activity

AMPK is one of the key cellular mediators required for maintaining cellular energy homeostasis. AMPK exists in multiple isoforms and it is a heterotrimeric complex composed of one alpha subunit (either α1 or α2), beta subunit (either β1 or β2), and gamma subunit (either γ1, γ2, or γ3) (113). Previous studies show that AMPK induced transcriptional upregulation of genes involved in mitochondrial metabolism require PGC-1α (114) and overexpression of AMPK increases PGC-1α expression (115). AMPK regulates PGC-1α activity via direct phosphorylation at threonine-177 and serine-538, and the effect of AMPK on increased expression on mitochondrial proteins and function is regulated via PGC-1α (62, 114). AMPK has also been shown to activate SIRT1, an enzyme which catalyzes deacetylation and activation of PGC-1α leading to mitochondrial biogenesis (116). Therefore, activation of the AMPK pathway is a promising approach to stimulate mitochondrial biogenesis in various disease conditions, such as sepsis, that negatively affect mitochondrial function.

Treatment with AICAR will induce mitochondrial biogenesis and function in skeletal muscle cells, an effect mediated through activation of SIRT1, which leads to deacetylation and activation of PGC-1α (64). In a murine cecal ligation and puncture (CLP) model, AICAR protected against the sepsis-induced derangements in cardiac architecture and dysfunction (65). AICAR treatment also protected against LPS-induced loss in muscle mass (66) and reduced pro-inflammatory cytokine production and sepsis-induced increases in markers of kidney and liver injury during CLP-induced sepsis. Inhibition of AMPK by compound C exacerbated sepsis-associated tissue injury (67).

Metformin, a clinically used biguanide anti-diabetic drug, improves mitochondrial function via activation of AMPK (68). The mechanisms leading to metformin-induced activation of AMPK include increased phosphorylation of AMPKα at Thr-172 and via inhibition of mitochondrial complex I leading to modulation of the cellular AMP/ATP ratio (69, 70). Studies by Suwa et al. recognized that metformin, a first line oral drug for the treatment of type 2 diabetes, increases PGC1-α and mitochondrial protein content in muscle through AMPK activation (71). Metformin has been shown to be protective in studies employing animal models of sepsis (117). During LPS- and CLP-induced sepsis, metformin protected against sepsis-induced injury in brain, heart, liver, and lung. These benefits were mediated through inhibition of oxidative stress and inflammation, reduced infiltration of neutrophils, maintenance of mitochondrial membrane potential, and preservation of mitochondrial function (72–76, 118). In humans, a metanalysis including five observational cohort studies found that pre-admission use of metformin was associated with decreased mortality among patients with sepsis and diabetes mellitus (77). This association warrants further study of causality and the mechanism behind this association to assess the therapeutic benefit of metformin during sepsis.

Despite the described benefits of AICAR and metformin in reducing inflammation and providing organ protection in experimental models of sepsis, little is known about the impact of these drugs on immune function in the septic or severely injured host, which provides fertile ground for future research.

#### 5-Hydroxytryptamine Receptor (5HT) Agonists

Specific agonists of the 5HT receptor family have been shown to induce mitochondrial biogenesis (119). 5HT is the chemical name for endogenous neurotransmitter serotonin. 5HT receptors are G-protein coupled receptors with serotonin functioning as its endogenous ligand. It remains to be determined if 5HT receptor agonists could provide therapeutic benefit to protect against sepsis-induced organ injury. Immune cells including macrophages, monocytes and T cells express 5HT receptors (120). Serotonin has been shown to augment the phagocytic capacity of murine peritoneal macrophages via 5HT_1A_ receptor subtype (78). Serotonin has also been shown to activate alveolar macrophages via 5HT_2c_ receptor leading to increased expression of the monocyte chemoattractant MCP-1 (79). Various studies have shown the stimulatory effect of serotonin on other immune cells including Natural Killer cells, dendritic cells, and T cells (120, 121). Studies evaluating the effect of serotonin and synthetic 5HT receptor agonists on mitochondrial biogenesis in leukocytes is currently lacking.

#### Peroxisome Proliferator-Activated Receptor (PPAR) Activators

PPARs are a class of nuclear receptors/transcription factors that are comprised of three isotypes including PPARα, PPARβ/δ, and PPARγ (122). PPARs are known to regulate various metabolic functions including triglyceride and lipoprotein metabolism, fatty acid synthesis, and oxidation and energy homeostasis to name a few (123). PGC1-α, the aforementioned transcription factor known for its role in mitochondrial biogenesis, also functions as a coactivator PPARγ (124). Thiazolidinediones are clinically used anti-diabetic drugs, which increase insulin sensitivity through activation of PPARγ (125). Rosiglitazone, a thiazolidinedione class drug, was shown to attenuate LPS-induced cardiac dysfunction and protect mitochondria leading to improved survival (80). Pioglitazone, another PPARγ agonist, has been shown to reduce inflammation and improve survival in a murine CLP and *Candida albicans-*induced sepsis (81, 82). Zingarelli et al. showed that treatment with PPARγ ligands, 15-deoxy-Delta(12,14)-PGJ(2) (15d-PGJ(2)), and ciglitazone attenuated inflammation, reduced excess neutrophil influx into various organs, decreased hypotension and improved survival through regulation of NF-κB and AP-1 signaling pathways using murine CLP model of sepsis (83). Other studies have also shown similar anti-inflammatory effects of synthetic PPARγ ligands including 15d-PGJ(2) and troglitazone on macrophages (84–86, 126). Fenofibrate, a known PPARα agonist used clinically for the management of dyslipidemia, reduced pro-inflammatory cytokines levels, promoted neutrophil recruitment to the site of infection and augmented bacterial clearance leading to improved survival in a murine model of *Salmonella typhimurium*-induced sepsis (87). The beneficial effect of fenofibrate was shown to be independent of PPARα but dependent on the preservation of neutrophil CXCR2 expression (87). Using another PPARα agonist, Crisafulli et al. demonstrated that clofibrate reduces LPS/IFNγ induced pro-inflammatory cytokine production in murine peritoneal macrophages (89). Treatment of pediatric burn patients with fenofibrate within the first week after burn injury has been shown to increase hepatic mitochondrial ATP production, maintain cytochrome c oxidase levels and citrate synthase activity along with improving insulin sensitivity, thereby indicating the therapeutic utility of fenofibrate-induced augmentation of mitochondrial function after burn injury (88). A study by Standage et al. showed that PPARα expression is decreased in the whole blood of pediatric sepsis patients and this correlated with the severity of sepsis outcomes and PPARα is required for maintaining optimal immune function during sepsis (127). In summary, PPAR agonists might have therapeutic potential in attenuation of sepsis induced inflammation and organ injury. However, the specific effect of various PPAR agonists on mitochondrial biogenesis and function in various organs and leukocytes in context of sepsis and trauma has not been investigated in detail and needs to be evaluated in future studies.

#### Phosphodiesterase (PDE) Inhibitors

Phosphodiesterases serve to hydrolyze cAMP and cGMP, increase levels of which reduces vascular tone, tightens endothelial junctions, and increases cardiac contractility. The cAMP-response-element-binding protein (CREB) is involved in transcriptional activation of PGC1α (128). In pediatric sepsis patients, treatment with PDE3 inhibitors increase both cAMP and cGMP levels and not only improve cardiac function (90, 129, 130) but also increase survival (131, 132). PDE4 inhibitors such as rolipram and Ro 20-1724 are selective for cAMP (133). Inhibition of PDE4 using Ro 20-1724 reduced systemic vascular resistance and improved cardiac and renal function in LPS model of sepsis in rats (91, 92). Treatment with rolipram improves renal blood flow, protects renal microcirculation and improves glomerular filtrate rate and renal function in a murine model of CLP-induced sepsis, even when administered 6 h after CLP (93). Rolipram treatment also improved renal and cardiac function leading to improved survival in septic rat pups (94). PDE4 inhibitors, rolipram and roflumilast, have been shown to reduce leukocyte-endothelial interactions which inhibits inflammatory cell influx, and reduce capillary leakage during LPS-induced inflammation (95, 96). Wollborn et al. showed that treatment with rolipram improves hepatic microcirculation and protects liver architecture in a rat model of LPS induced inflammation (97). Pharmacological agents such as rolipram and cilastozol which are specifically inhibit PDE4 and PDE3, respectively, and have been shown to increase CREB phosphorylation, upregulate PGC-1α expression and contribute to the induction of mitochondrial biogenesis (98, 99, 134). Future studies addressing the impact of PDE inhibitors on mitochondrial function in organs and leukocytes in context of sepsis and trauma are warranted.

#### Natural Products That Induce Mitochondrial Biogenesis

Resveratrol, a polyphenol compound found in grapes and red wine, has been shown to activate PGC1α and mitochondrial biogenesis through SIRT1 or AMPK signaling (135). Resveratrol upregulates PGC-1α, NRF1, NRF2 and Tfam leading to potentiation of mitochondrial biogenesis (100). In multiple studies using a CLP model of polymicrobial sepsis in rats, resveratrol treatment results in increased survival as well as decreased kidney injury associated with inhibition of NFκB (101, 102). In a similar model of pediatric sepsis-induced kidney injury in young rats, resveratrol was shown to activate NRF2 and protect from injury (103). Shang et al. report that resveratrol is protective in LPS-induced cardiomyopathy in rats also through inhibition of NFκB (104). In horses, however, Martin et al. showed that a 3 week course of resveratrol did not increase antimicrobial function or alter cytokine release profiles of *ex vivo* stimulated leukocytes (105).

Epigallocatechin gallate (ECGC), a natural compound found in tea, promotes cAMP dependent signaling and increases SIRT1 and consequently PGC1α (106). In murine LPS-induced endotoxemia, ECGC protected against acute lung injury and decreased proinflammatory cytokine production (108). ECGC has been shown to induce the NRF2 antioxidant response element through direct interaction with its inhibitor KEAP1 thereby leading NRF2 activation (107). NRF2, like PGC1α, is known to be involved in mitochondrial biogenesis. In the CLP model, ECGC attenuated hypotension and improved survival (109).

Estrogen receptors are known to regulate mitochondrial biogenesis, so it follows that phytoestrogens may also induce mitochondrial biogenesis and have protective affects in sepsis. A diet high in two phytoestrogens daidzein and genistein has been shown to increase PGC-1α expression, and these two compounds were separately shown to decreases proinflammatory cytokines in LPS-induced endotoxemia, and increase survival and bacterial clearance in CLP-induced sepsis respectively (110–112).

## Metabolic Reprogramming of Innate Leukocytes by Microbial Ligands

**Figure 1:**
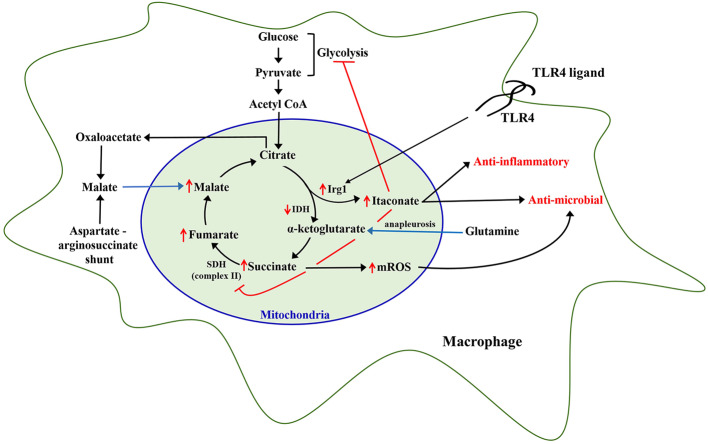
Metabolic reprogramming of leukocytes. Inflammatory stimulation of leukocytes, specifically monocytes and macrophages, with Toll-like receptor 4 (TLR4) ligands like lipopolysaccharide, has been shown to rewire mitochondrial metabolic pathways including upregulation of immunoresponsive gene 1 (Irg1) leading to increased itaconate generation, and increased accumulation of other TCA cycle metabolites including succinate, fumarate, malate, and citrate which continue to be replenished via additional pathways including glutamine anapleurosis and aspartate-arginosuccinate shunt. Itaconate produced by Irg1 inhibits succinate dehydrogenase, which causes an increase in mitochondrial reactive oxygen species (mROS). Itaconate and mROS augment antimicrobial capacity of leukocytes.

Stimulation of innate immune cells with microbial ligands such as LPS, peptidoglycan, or β-glucan reprograms their metabolism, which supports the increased physiological demands needed to augment antimicrobial capacity to combat invading infections (47, 136, 137). The reprogrammed phenotype of innate leukocytes manifests as distinct augmentation of glycolysis and mitochondrial tricarboxylic acid cycle flux and oxidative phosphorylation, as detailed below (Figure 1).

### Reprogramming of Glycolysis

Hard et al. discovered that immune macrophages, defined as those from peritoneal cavities of mice injected with bacteria, produced more lactate and consumed less oxygen than controls (138). Further investigations showed that macrophages stimulated with LPS manifest increased glucose uptake, an elevated glycolytic rate and augmentation of the pentose phosphate pathway (139, 140). These findings were reminiscent of the aerobic glycolysis noted by Warburg et al. in cancer cells, which preferentially utilize glycolysis, even in aerobic conditions that should favor oxidative phosphorylation as more energetically efficient (141). Aerobic glycolysis in macrophages in facilitated, in part, by stabilization of hypoxia-inducible factor (HIF)-1α. Early macrophage activation induces accumulation of succinate and itaconate, which are transported out of mitochondria in the cytosol where it acts to stabilize HIF-1α by impairing the activity of prolyl hydroxylases (142, 143). HIF-1α facilitates increased expression of numerous gene products that regulate inflammation including enzymes that promote glycolysis (140). Though this effect is notable in multiple types of murine macrophages, Vijayan et al. reported that LPS does not increase glycolysis in human PBMCs (144). Multiple purposes for this increase in glycolysis, over oxidative phosphorylation at the expense of energy efficiency, have been hypothesized. West et al. described that classically activated macrophages require mitochondrial reactive oxygen species for effective bacterial clearance (145). The contributions of mitochondrial complex I to ATP synthesis during oxidative phosphorylation may detract from mROS generation (145). As suggested in Viola et al., glycolysis may also be advantageous because it supplies biosynthetic intermediates important for rapid cellular adaptations, as well as NADPH through the pentose phosphate shunt, which is important for generation of ROS. The Warburg effect in macrophage activation is specific to the classical M1 phenotype, but not in alternatively activated M2 macrophages, which rely on oxidative phosphorylation (146). Interestingly, increases in oxidative phosphorylation and glycolysis occur in macrophages activated by the TLR4 agonist MPLA 72 h after exposure, resulting in a hybrid phenotype with metabolic characteristics common to both M1 and M2 macrophages (47).

LPS also induces the TCA cycle metabolite itaconate, in both murine and human macrophages (147) (Figure 1). It has been recently shown that itaconate inhibits glycolysis via inhibiting glycolytic enzymes aldolase A and glyceraldehyde-3-phosphate hydrogenase in RAW 264.7 macrophage cell lines (148, 149). Itaconate has also been shown to inhibit succinate dehydrogenase, which might reprogram citric acid cycle function and facilitate mROS generation due to reverse electron transport secondary to inhibition of SDH-dependent complex II (150).

### Reprogramming of Mitochondrial Metabolism

The majority of recent studies demonstrate significant alterations in the generation of TCA cycle intermediates upon TLR agonist-induced inflammatory stimulation of monocytes and macrophages. Studies from our laboratory, and others, show that citrate, itaconate, and succinate accumulate during metabolic rewiring of macrophages and monocytes (47, 140, 151, 152). Recent studies have elucidated a unique role for each of these metabolites in the context of cellular metabolic and antimicrobial functions.

Citrate is converted to α-ketoglutarate by isocitrate dehydrogenase (IDH) through the intermediate cis-aconitate. Michelucci et al., demonstrated that stimulation of macrophages with LPS leads to significant upregulation of immunoresponsive gene 1 (Irg1) enzyme, which catalyzes the production of itaconate from cis-aconitate in the mitochondria, thus diverting pyruvate-derived citrate production away from energy generation and toward production of itaconate (153). Jha et al., also showed that LPS induces downregulation of IDH and succinate dehydrogenase (SDH) function in macrophages leading to a significant accumulation of citrate and succinate (151). In line with this, studies from our laboratory show that MPLA treatment reduces TCA cycle flux between citrate and α-ketoglutarate at 24 h after stimulation in association with induction of Irg1 expression and large scale itaconate production (47). Therefore, it is evident that inflammatory stimulation of macrophages drives citrate toward production of itaconate. Itaconate has now been shown to be a critical regulator of macrophage and monocytic function after LPS stimulation. Intracellular itaconate concentrations of up to 8 mM have been shown in macrophages at 6 h after LPS stimulation (153), which subsequently steadily decline over time (152). There are multiple known downstream cellular effects of this dramatic increase in itaconate. First, itaconate inhibits mitochondrial complex II or SDH function in a dose-dependent manner leading to succinate accumulation (154), which is supported by the observation that Irg1 knockout macrophages do not accumulate succinate following LPS stimulation (151). The implications of succinate accumulation are discussed later. Itaconate also plays a major role in potentiating cellular anti-inflammatory and anti-oxidant effects through activation and nuclear translocation of NRF2via alkylation of KEAP1, a known physiological inhibitor of NRF2 (147). Through activation of NRF2, 4-octyl-itacoante (a cell permeable analog of itaconate) increases expression of key anti-inflammatory genes including heme oxygenase 1 and potently inhibits proinflammatory cytokine release (147). Macrophages lacking the Irg1 enzyme produce increased proinflammatory cytokines, including IL-6, IL-18, and IL-1β, in response to LPS relative to wild type macrophages and treatment with a cell permeable itaconate derivative decreases proinflammatory cytokines in response to LPS (147, 155).

Itaconate is also known to be secreted by macrophages into the extracellular milieu and have direct antibacterial effects (156). Itaconate competitively inhibits the microbial enzyme isocitrate lyase, a required step in the glyoxylate shunt, thereby limiting bacterial growth under nutrient poor conditions as occur at the site of infection (157). The microbial glyoxylate shunt bypasses two decarboxylation steps in the tricarboxylic acid cycle, facilitating the assimilation of carbon when only two-carbon sources such as ethanol or acetate are available (151, 158–160). Pathogens that have shown sensitivity to itaconate-induced microbial growth inhibition include *Mycobacterium tuberculosis, Staphylococcus aureus, Legionella pneumonia, Acinetobacter baumanii*, and *Salmonella enterica* (153, 161, 162). Therefore, itaconate affects cellular metabolism and affords anti-inflammatory and anti-microbial protection upon inflammatory activation of immune cells. As such, our knowledge of the role of itaconate is currently limited to macrophages and monocytes, and future studies addressing its effects on other leukocytes such as neutrophils and dendritic cells will shed more light on the novel aspects of this critical metabolite. Nonetheless, based on studies, therapeutic utility of itaconate to protect against life-threatening infections and sepsis merits further investigation.

Succinate is another TCA cycle metabolite that significantly accumulates in LPS-stimulated macrophages and monocytes (150, 152, 163). Succinate is the principal substrate for succinate dehydrogenase, which not only participates in the TCA cycle but also in ETC complex II. Oxidation of succinate to fumarate results in reduction of FAD+ and ultimately Coenzyme Q, which continues in the ETC via complex III and IV, leading to ATP generation via ATP synthase (16). Itaconate-induced inhibition of SDH and facilitation of glutamine anapleurosis are the major sources of intracellular succinate accumulation upon LPS stimulation of macrophages (150, 151). High levels of succinate and succinate dehydrogenase activity are associated with inducing a pro-inflammatory phenotype in innate leukocytes as result of succinate-mediated hypoxia inducible factor α (HIF-1α) stabilization, increased mitochondrial ROS generation, and protein succinylation (137, 163). LPS-induced succinate accumulation is associated with stabilization of HIF-1α, leading to increased IL-1β production and inflammation (140, 164). Rapid oxidation of increased succinate to fumarate by SDH requires CoQ, which is consumed under LPS stimulation, thereby driving reverse electron transport leading to a substantial generation of mitochondrial ROS (165). Although uncontrolled generation of mitochondrial ROS can have deleterious effects on cellular functions, it has also been shown to play an important role in microbial clearance (145). However, further studies are needed to establish the antimicrobial role of SDH-generated ROS in *in vivo* models of infection.

Inflammation-induced increases in intracellular accumulation of citrate also affects cellular metabolism and functions. Activated macrophages accumulate citrate due to decreased isocitrate dehydrogenase activity (47, 151). De Souza and colleagues recently demonstrated that LPS-mediated increase in IFN-γ limits isocitrate dehydrogenase activity in an autocrine manner in macrophages, implying a role for IFN-γ in LPS-mediated increase in citrate levels (166). Accumulated citrate is not only converted to itaconate (153) in the mitochondria but also transported from the mitochondria into the cytosol via mitochondrial citrate carrier (CIC) (167). Increased CIC and cytosolic citrate has been shown to fuel the LPS-induced generation of pro-inflammatory mediators such as nitric oxide, ROS, and prostaglandins in macrophages (168). Our studies also show that MPLA-stimulated citrate transported into the cytosol is ultimately converted to malate and pyruvate, and the cytosolic malate replenishes mitochondrial oxaloacetate pools to further fuel a sustained increase in mitochondrial TCA cycle flux (47). Importantly, these alterations in citrate metabolism are associated with a sustained augmentation of mitochondrial density and oxygen consumption, along with increased macrophage phagocytic capacity (47). Therefore, citrate accumulation not only plays an important role in fueling acute inflammation but also potentiates a sustained increase in TCA cycle flux and antimicrobial functions, which need further evaluation.

### Evidence for Metabolic Reprogramming in Murine and Human Sepsis Studies

The majority of studies demonstrating the effect of inflammatory activation on metabolic reprogramming of innate leukocytes such as macrophages and monocytes have been performed *in vitro*. Corroborating the changes described in the *in vitro* studies described above, metabolic reprogramming of innate immune cells in response to TLR activation has also been observed in some *in vivo* murine and human studies. Sterile endotoxemia (LPS administration) in mice causes peritoneal macrophages to more than double glucose uptake, suggesting an increase in glycolysis in this model (169). Functionally, monocytes from septic patients were found to have increased basal glycolysis compared to healthy controls (170). Shalova et al. performed a gene ontology analysis to compare monocytes from septic patients relative to healthy controls, and reported that the top 10 most significantly downregulated gene clusters were all related to cellular metabolism (171). Consistent with this, Cheng et al. found diminished glycolysis and oxidative phosphorylation in peripheral blood mononuclear cells (PBMCs) in septic patients with immunoparalysis as compared to control subjects (31). Genome-wide microarray analysis of PBMCs from patients with both bacterial and fungal sepsis in this study identified that genes for oxidative phosphorylation and glycolysis were both increased along with evidence of mitochondrial dysfunction pathways, suggesting that immune cell metabolism is significantly affected during sepsis. Further studies to separate the adaptive from the pathogenic changes in leukocyte metabolism could guide the development of therapies to augment or suppress these metabolic changes. For example, a study by Pan et al. demonstrated that a known anti-inflammatory compound, deoxyelephantophin, both blocks LPS-induced glycolytic increase and protects mice against endotoxemia (172).

There are limited *in vivo* studies analyzing the effect of sepsis on alterations of specific mitochondrial TCA cycle intermediates during sepsis. A murine study by Chao et al. employing scrub typhus infection demonstrated a 60-fold increase in plasma itaconate levels at 10 days after infection (173). A clinical study by Meiser et al. reported absence of any detectable itaconate in the plasma and urine of septic patients, in which the authors concluded that itaconate may not be a suitable systemic biomarker for predicting sepsis outcomes (174). That study evaluated the levels of itaconate at a single time point among sepsis patients and failed to elaborate on the clinical condition of patients during sample collection and the exact time point for collection. A recent study by Beloborodova et al. detected low concentrations of itaconic acid (0.5–2.3 μM) in the plasma of septic shock patients collected within 24 h and none was detected in patients at later stages of sepsis (175). The levels of succinate were higher in the late stage sepsis patients as compared to early stage, but lower than the control healthy group. It must, however, be noted that the early and late stage sepsis patients included in this study were entirely different patient cohorts and the authors do not report the changes in plasma itaconate levels as sepsis progressed in each septic patient subset. It is critical to follow septic patients and study the alterations in itaconate levels at various time points after sepsis induction to derive a definitive conclusion for the use of itaconate as a biomarker for sepsis outcomes or for supporting itaconate's use for therapeutic purpose to combat sepsis. Future studies evaluating sepsis-induced alterations in the levels of mitochondrial metabolites would be critical to further the field of metabolic reprogramming toward discovery of novel therapeutics to protect against infections and sepsis.

## Innate Immune Memory and Trained Immunity

**Figure 2:**
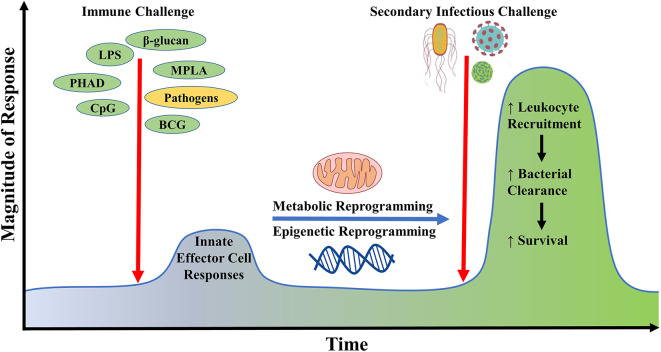
Generation of innate immune memory using microbial ligands. Initial challenge with microbial ligands such as lipopolysaccharide, monophosphoryl lipid A, CpG, β-glucan potently stimulates host innate effector immune responses in cells such as neutrophils, monocytes, and macrophages, leading to the reprogramming of their metabolic and epigenetic status. Upon re-exposure of the initially primed host with a secondary inflammatory stimulus or infectious challenge, there occurs a heightened innate immune response against invading microbes via increased immune cell recruitment leading to improved microbial clearance and survival. This phenomenon is termed as innate immune memory.

Classically, the role of the innate immune system is to recognize pathogens and mount a non-specific yet rapid response, whereas immunological memory has been traditionally considered a unique hallmark of the adaptive immune system. However, recent studies indicate that innate immune cells adapt upon exposure to a pathogen or pathogen-derived ligand, triggering augmentation of cell physiology and antimicrobial functions which allows for robust responses to a subsequent challenge either by the same or different pathogen (176). This phenomenon by which innate antimicrobial efficiency is increased due to the priming effect of prior exposure is termed “innate immune memory” or “trained immunity” (Figure 2). This immunoregulatory process confers host resistance to infection in plants and invertebrates that do not have adaptive immunity but also in mammals (177). The cell type (myeloid, natural killer, and innate lymphoid cells), stimuli (pattern recognition receptors and cytokines), genetic mechanism (epigenetic rewiring), and time scale (persisting weeks to months) are unique to innate immune memory, independent of those involved in classical immunological memory (178). An important player in health and disease, trained immunity may also serve as an innovative therapeutic strategy for protecting vulnerable patients from life-threatening infections in the future.

### Metabolic Reprogramming and Innate Immune Memory

Recent findings strongly indicate that metabolic reprogramming is a key process underlying development of innate immune memory. Several studies have revealed that expression of key pro-inflammatory proteins and an effective immune response relies on intact mitochondrial respiration (179, 180), and the study of the metabolic demands of mounting an immune response has been a topic of increasing interest (181). It has become widely appreciated that metabolism dynamics regulate innate immunity via production of metabolite intermediates which influence cellular phenotype and function (182). β-glucan immunomodulation has been associated with upregulated glycolysis in trained macrophages (183) and monocytes (184), likely to support pro-inflammatory macrophage antimicrobial functions (182, 185). This has been shown to be dependent on a shift from oxidative phosphorylation toward glycolysis through an Akt/mTOR/HIF-1α dependent pathway (183, 186). We recently reviewed regulation and function of HIF-1α in myeloid cells (187). On the other hand, TLR ligands (such as LPS, MPLA, and CPG) increase aerobic glycolysis in concert with increased antimicrobial functions (such as respiratory burst and phagocytosis) as well as induce mitochondrial biogenesis and increased oxidative metabolism (47). These metabolic alterations allow immediate leukocyte activation, cytokine secretion, and a more effective innate immune response to infection (46, 47, 188, 189). Our study using HIF-1α deficient macrophages demonstrated that HIF-1α is required for these metabolic alterations (46). Another study from our group showed that the inhibition of mTOR, which stabilizes HIF-1α, diminishes the protective response of TLR4 ligands (47).

Despite the apparent benefits of inducing innate immune memory, reprogramming of leukocyte oxidative metabolism could be a double-edged sword. As noted above, current research indicates that priming the immune system with microbial ligands at doses that do not cause damaging systemic inflammation induces protective immunity in association with an increase in leukocyte oxidative metabolism (47, 48). It appears that the heightened metabolic state induced under those conditions is utilized to facilitate augmented leukocyte antimicrobial functions such as phagocytosis, oxidative burst, and microbial killing. However, in cases of tissue injury, reprogrammed leukocytes could funnel energy to drive hyperinflammation. A recent paper by Di Gioia and colleagues showed that oxidized phospholipids derived from 1-palmitoyl-2-arachidonyl-sn-glycero-3-phosphorylcholine (oxPAPC) can induce increased leukocyte oxidative metabolism and hyperinflammation, especially in the presence of microbial ligands such as LPS (190). Oxidized phospholipids are damage associated molecular patterns (DAMPS) that are released following tissue injury. Di Gioia and colleagues reported that oxPAPC and LPS strongly drive production of pro-IL-1β in macrophages, which is cleaved and secreted as the mature protein upon activation of the inflammasome by DAMPS such as ATP (190). However, the ramifications of these alterations in models of acute inflammation remain to be fully elucidated since a study by Chu and colleagues showed that oxPAPC inhibits non-canonical inflammasome activation and is protective in an experimental model of septic shock (191).

## Innate Immune Memory—a Novel Therapeutic Target to Protect Against Infections and Sepsis

The non-specific protection conferred by trained immunity lends itself to an exciting novel therapeutic approach by which patients could be primed and protected from a wide array of infections thus preventing sepsis and subsequent mortality. Several microbial ligands have immunomodulatory potential, most notably, TLR and dectin-1 agonists. Rowley first reported in 1956 that priming mice with the TLR4 agonist lipopolysaccharide (LPS), a structural component of the cell wall of Gram-negative bacteria, conferred host protection to subsequent exposure to Gram-negative pathogens (192). Following this discovery, it has been found that LPS challenge protects against a wide array of pathogens, including fungal (193), Gram-positive *Staphylococcus aureus* (194), and several Gram-negative pathogens, including *Escherichia coli* (192), *Salmonella enterica serovar typhimurium* (195), and *Pseudomonas aeruginosa* (196, 197), as well as polymicrobial sepsis (198). Priming with LPS induces enhanced bacterial clearance (196, 199) and leukocyte recruitment (194, 200).

Leukocytes primed with LPS can also be described as not only trained, but also “endotoxin tolerant,” which is defined by an attenuated pro-inflammatory response upon secondary challenge with the stimulus. A body of literature suggests that the phenomenon of endotoxin tolerance is a state of immunoparalysis during which the host is more susceptible to infection (201, 202), and results in poorer patient outcomes (203–206). However, the clear relationship between endotoxin tolerance and susceptibility to later infections has not been established. In fact, our group recently demonstrated that the cytokine response to LPS is not indicative of antimicrobial immunity (46), and a body of literature illustrates that altering proinflammatory cytokines during infection has had no protective benefit (207–210) thereby bringing into question whether proinflammatory cytokine levels are an essential element in determining immune competence.

### TLR4 Agonist-Induced Innate Immune Memory and Protection Against Infection

As LPS is toxic to humans, experimental studies have progressed to investigate other agonists that confer this attractive phenotype of host resistance to infection after priming. Intriguingly, prophylactic administration of the vaccine adjuvant MPLA, which is derived by cleaving the C1 phosphate group from lipid A and is 100-fold less toxic than LPS (211–213) improves bacterial clearance, attenuates physiologic dysfunction, induces leukocyte expansion and recruitment to sites of infection, enhances antimicrobial functions, and profoundly improves survival during infection with a wide array of clinically relevant pathogens (47, 188, 214–217). TLR4 is unique among TLRs as it can generally signal through both the myeloid differentiation primary response gene 88 (MyD88)-dependent and the TIR-domain-containing adapter inducing interferon-β (TRIF)-dependent pathways. A study of human neutrophils, however, revealed that TLR4 activation by LPS does not activate the TRIF-dependent pathway in neutrophils, postulated to be due to neutrophil's more prominent role in bacterial responses compared to viral (218). Our group is investigating the relative contribution of these pathways in TLR-mediated trained innate immunity, and has shown that MyD88 deficient mice fail to augment leukocyte recruitment or G-CSF production in response to infection following priming with MPLA, both of which are known to play a critical role in MPLA-mediated protection (188, 189). Further, the MyD88-selective TLR9 agonist CpG oligodeoxynucleotide (CpG) preserves physiologic function and improves bacterial clearance following infectious challenge with *Pseudomonas aeruginosa* (46). CpG similarly provided protection in a model of intracerebral *Escherichia coli* (219), which implies that TLR-mediated resistance to infection is dependent on MyD88 signaling.

TLR4 agonist-induced antimicrobial properties are independent of antibiotic therapy. This is of particular importance due to the current rise in global antibiotic resistance (220–222). The rate of antibiotic resistance has been far exceeding the rate of new antibiotic class development, and current market trends suggests pharmaceutical companies will not be able to support new antibiotic development programs (220, 223). Thus, there is an increasing need for novel antimicrobial therapeutic strategies, lending to the possibility of adopting agents that induce trained immunity as independent or adjunct antimicrobial therapeutic agents. Several synthetic ligands that target TLRs and dectin-1 are under development. Novel synthetic phosphorylated hexaacyl disaccharides (PHADs), which target TLR4, are equipotent with MPLA as agents to augment antimicrobial immunity and have strong potential to be developed into drug candidates (48). PHADs are synthesized *de novo* and are currently under investigation as immunopotentiating agents (48, 213). The antimicrobial functions of PHADs are linked to the increased recruitment of innate leukocytes to the sites of infection and augmentation of their antimicrobial activity.

### Therapeutic Utility of Other Microbial Ligands

The class of TLR agonists that have strong potential for clinical translation extend beyond TLR4 ligands. The TLR9-selective agonist CpG oligodeoxynucleotide (CpG-ODN) is a short single-stranded synthetic bacterial DNA molecule that has been shown to confer host resistance to an array of pathogens including the parasite *Leishmania major* (224), the Gram-negative pathogens *Francisella tularensis* (225), *Pseudomonas aeruginosa* (226), and *Burkholderia pseudomallei* (227–229), Gram-positive *Listeria monocytogenes* (230), and viral HSV infections (231). Further, CpG-ODN also has promise as a vaccine adjuvant (232) and antitumor therapeutic (233, 234). There are several classes of CpG-ODN based on their variety of sequence and structure which elicit specific immunomodulatory profiles (232). Unlike TLR4, which signals through both MyD88- and TRIF-dependent pathways, activation of TLR9 triggers MyD88-dependent signaling alone. CpG-mediated host protection to infection seems to be dependent on downstream induction of Th1-type immune response, specifically the production of Interferon-β (224, 235). Further work is necessary to define the cellular and molecular underlying mechanisms by which CpG boosts antimicrobial responses and protects against infection.

Other microbial ligands and infections themselves can induce innate immune memory and enhance antimicrobial functions through different signaling mechanisms. β-glucans are structurally diverse polysaccharide components found mainly in fungal cell walls that are key pathogen-associated molecular patterns that trigger an immune response and are the quintessential inducers of trained immunity (236). Glucans are potent immunomodulators that augment host resistance against Gram-negative [*Escherichia coli*; (237, 238)], Gram-positive (*Staphylococcus aureus)* (239, 240), fungal [*Candida albicans*; (241)], and parasitic (*Leishmania braziliensis)* (242) infections. Glucan binds Dectin-1, which triggers downstream Raf-1/Akt-dependent signaling to augment phagocytosis, ROS production, microbial killing, and cytokine production (243–245). Further, glucan has been shown to decrease infectious complications in high risk surgical patients (246). The biological mechanisms underlying the immunomodulatory effects of glucan remain to be fully understood but glucan strongly induces metabolic reprogramming and epigenetic changes that alter gene expression and augment leukocyte function (236). Interestingly, trained immunity can also be induced by Bacillus Calmette-Guerin (BCG), which has conferred resistance to *Schistosoma mansoni* (247) and *Candida albicans* (248) infections in mice. These studies found that BCG-primed macrophages show increased phagocytosis and ROS production and improved clearance of pathogens. Epidemiological studies show that BCG, among other vaccines such as measles and oral polio vaccine, confer beneficial protective effects to unrelated pathogens in humans (249–251). Furthermore, evidence suggests that certain viral infections, such as malaria (252) and murine cytomegalovirus (253, 254), and parasitic infections [*Nippostrongylus brasiliensis*; (255)] induce a state of cross-protection to different pathogens through increased innate antimicrobial efficiency.

## Conclusions

Here, we have reviewed the impact of sepsis on the mitochondrial function of innate leukocytes, and potential therapeutic strategies for reprogramming leukocyte metabolism to induce innate immune memory and restore host immune competency. Studies in both animal sepsis models and human septic patients reveal significant mitochondrial dysfunction in various organ systems, which correlates with sepsis severity and outcomes. In particular, sepsis-induced mitochondrial dysfunction in leukocytes is a key driver of impaired immune responses leading to increased susceptibility to secondary infections in septic patients. Studies show that early recovery of mitochondrial function in leukocytes correlates with improved septic patient outcomes.

TLR agonists are a class of microbial ligands with attractive immunomodulatory properties. Recent studies demonstrate that TLR agonists can mediate non-specific protection against infection with protective effects lasting up to 2 weeks, independent of the adaptive immune system. This induction of apparent innate immune memory is mediated by TLR agonist-induced metabolic reprogramming of leukocytes. The altered metabolic phenotype is characterized by increased glycolysis, oxidative phosphorylation, and intra-cellular concentrations of key metabolic intermediates such as itaconate and succinate, which influence cellular antimicrobial and anti-inflammatory functions. Current studies show that administration of drugs such as TLR ligands which boost leukocyte oxidative metabolism days prior to infectious challenge improve survival. Therefore, pre-treatment of critically, who are at risk for acquiring life-threatening infections, with immunomodulators that induce metabolic reprogramming and innate immunity might augment host resistance to infection and improve survival. *In vitro* data demonstrates that oxidative metabolism is boosted ~3 days after treatment. Though it is impossible to predict exactly which patients will face an infectious challenge when, patients at risk for hospital acquired infections could be dosed at admission or prior to an event that may lead to infection, such as abdominal surgery. A recent study by Casilag et al. shows that combination therapy with MPLA significantly augmented the efficacy of antibiotics leading to reduced bacterial burden and improved survival in a murine model of bacterial pneumonia, even when administered after induction of pneumonia (256). Therefore, treatment with immunomodulators such as TLR agonists and others may also be beneficial later in the course of sepsis to augment host innate immunity and improve outcomes.

With the increasing development of antimicrobial resistance, host-directed immunotherapies offer a promising approach to combat the risk of deadly infections in critically ill and injured patients. Immunomodulatory strategies aimed at augmenting host immunity provide a means of mediating sustained broad protection against a variety of common nosocomial pathogens. This review highlights the prospect of developing microbial ligands as novel therapeutics with the aim of augmenting leukocyte mitochondrial function and inducing innate immune memory for protection against life-threatening infections in critically ill patients.

## Author Contributions

All authors contributed toward writing of the manuscript sections and conceptualization of figures. NP and ES critically revised the manuscript for important intellectual concepts. All authors have read and approved the submitted version.

## Conflict of Interest

The authors declare that the research was conducted in the absence of any commercial or financial relationships that could be construed as a potential conflict of interest.
